# Ventral Hippocampal Kappa Opioid Receptors Mediate the Renewal of Fear following Extinction in the Rat

**DOI:** 10.1371/journal.pone.0058701

**Published:** 2013-05-10

**Authors:** Sindy Cole, Rick Richardson, Gavan P. McNally

**Affiliations:** School of Psychology, The University of New South Wales, Sydney, Australia; McLean Hospital/Harvard Medical School, United States of America

## Abstract

The hippocampus is part of a neural network which regulates the renewal of fear following extinction. Both the ventral (VH) and dorsal (DH) hippocampus have been shown to be necessary for renewal, however the critical receptors and neurotransmitters mediating these contributions are poorly understood. One candidate mechanism is the kappa opioid receptor (KOR) system, which has been implicated in fear learning and anxiety. Here we examined the effect of the KOR antagonist norbinaltorphamine hydrochloride (norBNI), infused into either the VH or DH, on the renewal of extinguished fear. We found that an infusion of norBNI into the VH significantly reduced the relapse of fear on test compared to that seen in saline controls (Experiment 1), while similar infusions of norBNI into the DH had no effect on renewal (Experiment 2). These findings show that hippocampal KORs are involved in fear renewal and also identify a dissociation in the contribution of VH and DH KORs to the expression of renewed fear.

## Introduction

Animals will readily learn to fear a neutral cue such as a tone, following pairings of that cue with an aversive outcome like footshock. This learned fear of the tone (conditioned stimulus; CS) can be reduced by extinction, a process where repeated presentations of the CS alone cause a decline in the fear responses usually elicited by that CS. Because extinction forms the basis of exposure-based therapies, which are the most widely used treatments for anxiety disorders, it is imperative to understand the underlying mechanisms of extinction. Critically, extinction does not completely erase the original learning, but at least in part acts to form a new inhibitory memory which masks expression of the original fear memory and reduces fear responses [1]. As such, a number of factors can lead to retrieval of the original fear memory and the relapse of fear. For example, exposure to the CS outside of the extinction context causes the “renewal” of fear [2]. Understanding the neural mechanisms of such renewal may provide insight into improving the long term outcomes of exposure therapy.

The hippocampus is one structure important for contextual learning and memory [3] and is also critical for fear renewal. Although the dorsal hippocampus (DH) and the more caudal ventral hippocampus (VH) differ substantially in regards to neuronal connectivity and function [4], both have been implicated in renewal. For example, initial studies demonstrated that reversible inactivation of the DH significantly impairs renewal [5]. More recently the VH has also been implicated in mediating fear renewal, particularly through its direct projections to medial prefrontal cortex (mPFC) and basal amygdala (BA) [6], [7], [8]. For example, Herry and colleagues [9] showed that BA neurons active during renewal receive projections from VH, and disconnecting VH from either the mPFC or BA [10], or lesioning VH [11], disrupts renewal. Although this basic circuitry of fear renewal has begun to be elucidated, the mechanisms through which this circuit functions remain poorly understood.

Recently, there has been increasing interest in the potential therapeutic merit of the kappa opioid receptor (KOR) system in a range of psychiatric pathologies, including anxiety and depression. This is due in part to studies demonstrating the anxiolytic and anti-depressant-like properties of KOR antagonists like norBNI [12], [13]. Furthermore, central norBNI impairs the acquisition of fear conditioning [14]. We recently reported that intracerebroventricular administration of norBNI also impairs the expression of fear renewal [15], however the exact neuroanatomical locus of this action is presently unknown. Because KORs are expressed in moderately high levels in the hippocampus [16], here we studied a potential role for hippocampal KORs in the renewal of extinguished fear. We found a dissociation in the contribution of hippocampal KORs, such that KORs in the VH, but not the DH, mediate the renewal of fear.

## Materials and Methods

### Subjects

Experimentally naive male Wistar rats (250–350 g) obtained from Monash Animal Services (Gippsland, Victoria, Australia) were housed in groups of 8 in plastic cages with *ad libitum* access to food and water. The colony room was maintained at 21°C on a 12 h light/dark cycle (lights on 7 am) with all behavioral testing conducted during the light phase of the cycle. Rats were handled each day for 3 days prior to surgery to habituate them to the experimenter.

### Ethics statement

All experiments were carried out in accordance with the recommendations in The Australian Code of Practice for the Care and Use of Animals for Scientific Purposes (7th Edition). The procedures were approved by the Animal Care and Ethics Committee at The University of New South Wales (Permit Number 07/86B). All surgery was performed under ketamine/xylazine anesthesia, and all efforts were made to minimize suffering and the number of animals used.

### Surgery and histology

Rats were injected intraperitoneally with 1.3 ml/kg of the anesthetic ketamine (Ketapex; Apex Laboratories) at a concentration of 100 mg/ml, with 0.3 ml/kg of the muscle relaxant xylazine (Rompun; Bayer) at a concentration of 20 mg/ml, and subcutaneously with 5 mg/kg of the analgesic carprofen (Rompun; Bayer). Each rat was placed in a stereotaxic apparatus (Model 900, Kopf) and the skull was adjusted so that bregma and lambda were in the same horizontal plane. 26-gauge guide cannulae (Plastics One) were implanted bilaterally through holes drilled in the skull into either the VH (anteroposterior −6.3 mm; mediolateral ±5 mm; dorsoventral −5 mm) or the DH (anteroposterior −3.8 mm; mediolateral ±2.5 mm; dorsoventral −2.5 mm). Co-ordinates were determined from published studies targeting these hippocampal regions [17], [18]. The guide cannulae were fixed in position with dental cement and anchored by jeweler's screws. A dummy cannula to prevent occlusion was kept in each guide cannula at all times except during microinjections. Immediately after surgery, rats received prophylactic intramuscular injections of 0.3 ml of a 300 mg/ml solution of procaine penicillin and 0.3 ml of 100 mg/ml cephazolin sodium. Rats were allowed 5 days to recover from surgery, during which time they were monitored and weighed daily.

At the conclusion of each experiment rats were given an overdose of sodium pentobarbital. The brains were removed, frozen (−20°C), and sectioned coronally (40 µm) using a cryostat. Every second section containing cannula tracts was dry mounted onto glass microscope slides, stained for nissl bodies with cresyl violet, and coverslipped. Cannula placements were verified using light microscopy and mapped using the atlas of Paxinos and Watson [19].

### Microinfusions

For intracranial infusions, the dummy cannulae were removed and cannula injectors were connected via polyethylene-50 tubing to a 10 µl glass Hamilton syringe mounted on an infusion pump (KD Scientific). The injectors projected an additional 1 mm ventral to the tip of the guide cannulae. Norbinaltorphamine hydrochloride (norBNI: Tocris, Bristol, England) was dissolved in pyrogen-free saline and given at a dose of either 5 µg or 2.5 µg infused into each hemisphere, resulting in total doses of 10 µg and 5 µg respectively. Control infusions consisted of the pyrogen-free saline. All solutions were infused at volumes of 0.5 µl over 2 min, with the injectors left in place for an additional 2 min to permit diffusion. Following microinfusion the dummy cannulae were reinserted and the rats were returned to their home cage.

### Apparatus

The present experiments were conducted in two sets of four chambers located within two different rooms in the laboratory which differed in their visual, olfactory, and tactile properties. The chambers which served as context A (30 cm [length] ×30 cm [width] ×30 cm [height]) were constructed entirely of clear Perspex and the floor in each chamber consisted of stainless steel rods 2 mm in diameter spaced 10 mm apart (center to center) which were wired to a constant-current shock generator. Each chamber was located in a sound attenuating cabinet painted black and stood 2 cm above a tray of corncob bedding (Able Scientific). Illumination was provided by a 24 watt house light. In the chambers which served as context B (24 cm [length] 30 cm [width] 21 cm [height]) the top and rear walls as well as the front hinged door and the floor were constructed of clear Perspex and the side walls were made of stainless steel. Each chamber was located in a sound attenuating cabinet painted white, illuminated with an infrared LED and stood 2 cm above a tray of corncob bedding (Able Scientific). Dilute peppermint essence was placed in the bedding beneath the context B chambers. All cabinets were equipped with a ventilation fan (providing constant background noise), speaker, and digital video camera connected to a digital multiplexer and DVD recorder in an adjacent laboratory to record each session. Between each use, the chambers were cleaned with water, and where appropriate, the bedding beneath the floor was changed and essence was replaced. The CS was a 20 s 82 dB (A Scale), 750 Hz tone (0.1 s rise and fall). The footshock US was a 0.5 s, 0.7 mA unscrambled AC 50-Hz shock from a constant current generator delivered to the grid floor of each context A chamber. The stimuli used for conditioning were controlled by computer (LabView, National Instruments). Contexts were not counterbalanced due to the need for the training (A) context to possess the grid floor to allow footshock delivery.

### Procedure

**Figure 1:**
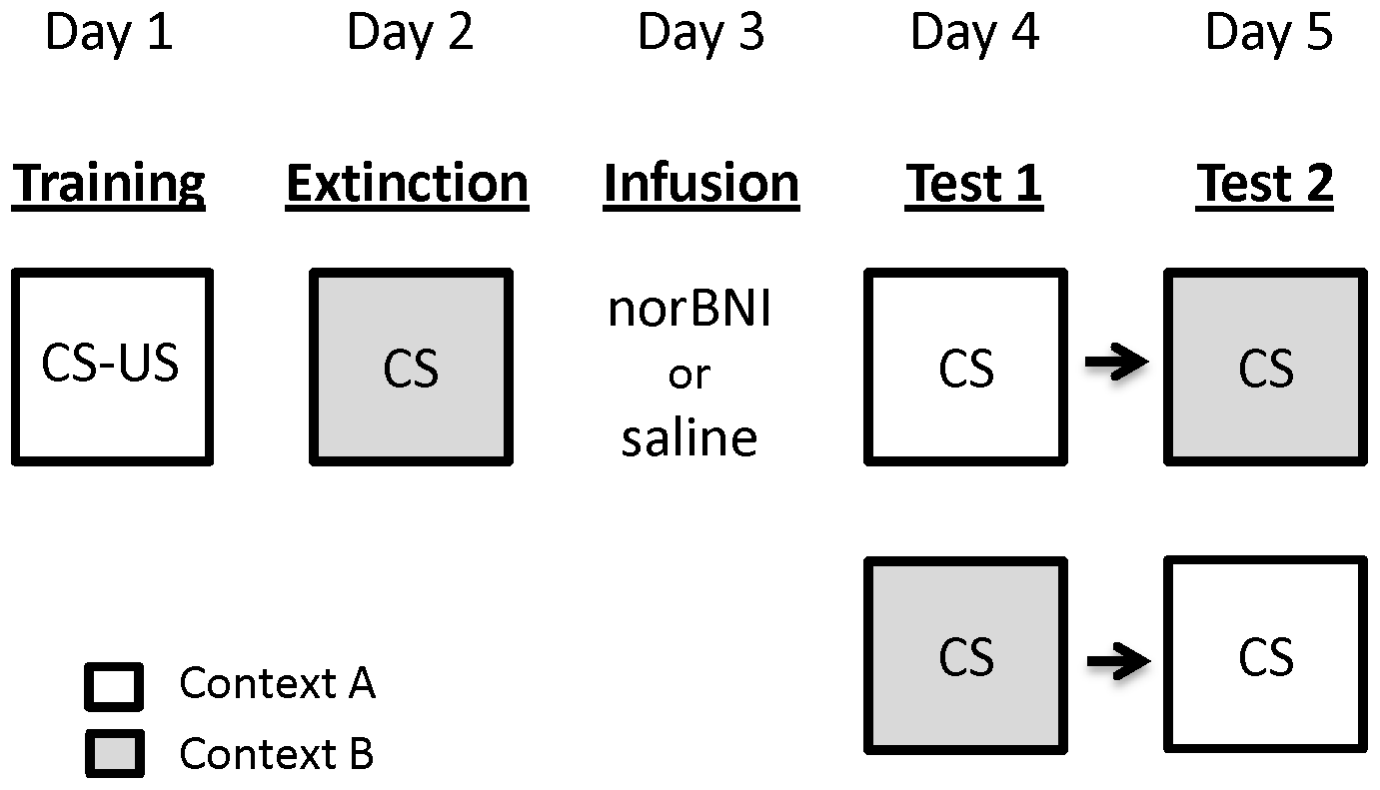
Experimental design. CS-US denotes pairings of a tone with footshock, while CS denotes presentations of the tone alone. In Experiment 1 norBNI (10 µg or 5 µg) or saline was infused into the ventral hippocampus. In Experiment 2 norBNI (10 µg) or saline was infused into the dorsal hippocampus. Animals were tested for responses to the tone in both contexts in a counterbalanced order. norBNI was infused 24 hours prior to the beginning of test due to the slow onset latency of the drug.

Conditioning, extinction, and test were identical across experiments (Figure 1). On Day 1 rats received fear conditioning in context A. After a 10 min pre-CS period all rats received 3 CS-US pairings with a 3 min inter-trial interval (ITI). On Day 2 rats received extinction training in context B. After a 2 min pre-CS period rats received 18 CS alone presentations with a 3 min ITI. On Day 3 rats received infusions of norBNI or saline as per group allocation and were then returned to their home cage. In Experiment 1 animals received either saline (n = 8), 5 µg of norBNI (n = 7), or 10 µg of norBNI (n = 8) into the VH. In Experiment 2 animals received either saline (n = 8) or 10 µg of norBNI (n = 7) into the DH. Infusions were administered 24 hours prior to the beginning of test due to studies demonstrating that norBNI has a slow onset [20] and extended duration of action (>2 weeks) [21]. On Days 4 and 5 all rats were tested for fear reactions to the tone CS in both context A and context B in a counterbalanced order. On test days rats were placed in the appropriate chamber where after a 2 min pre-CS period they received 4 presentations of the tone CS with a 2 min ITI. Other than during explicit training and infusion procedures, all animals remained in their home cages for the duration of the experiment.

### Scoring and statistics

Freezing, defined as the absence of all movement other than that required for respiration, was used as the measure of fear. Behavior during extinction and test was scored every 2 s as either freezing or not freezing by a trained observer unaware of group allocation. The number of freezing observations were summed and converted to a percentage. These data were analyzed using mixed analysis of variance (ANOVA) with planned orthogonal contrasts controlling the error rate at .05 for each contrast tested. Responding across extinction was collapsed into six extinction blocks, with each block consisting of 3 CS presentations. Extinction data were analyzed assessing the between-subjects differences in drug treatment and the within-subjects differences across blocks. Responding at test was analyzed assessing the between-subjects differences in drug treatment and the within-subjects differences in CS-responding between the training (A) and extinction (B) contexts. In Experiment 1 pre-CS freezing prior to extinction, test in context A, and test in context B, were analyzed using one-way ANOVAs. In Experiment 2 pre-CS freezing prior to extinction, test in context A, and test in context B, were analyzed using independent samples *t* tests. Standardized 95% confidence intervals were constructed for effect sizes. The statistical packages PSY [22] and SPSS (v.20) were used for all analyses.

## Results

### Experiment 1: Effect of KOR antagonism in the VH on the renewal of fear

**Figure 2:**
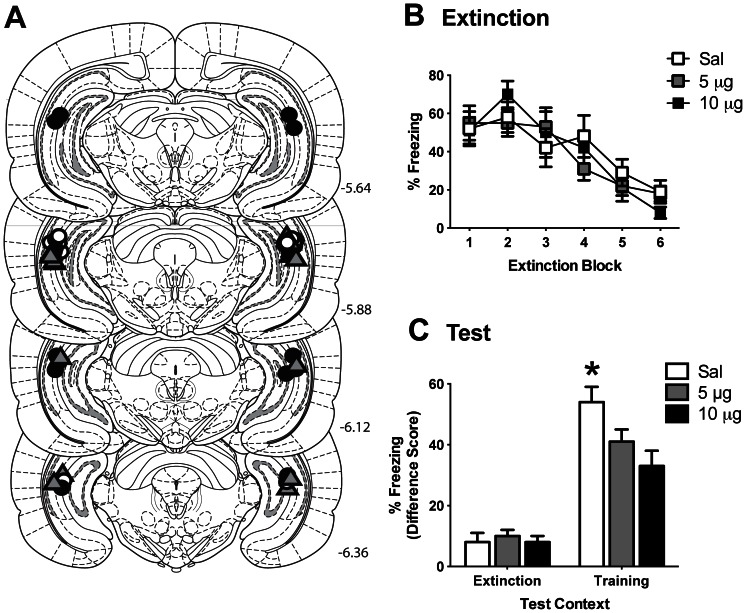
Infusion of norBNI into the ventral hippocampus (VH) reduces the renewal of fear. **A**. Microinfusion cannula placements in the VH for all rats included in Experiment 1 mapped onto coronal sections from the atlas of Paxinos and Watson (2007). Numbers indicate distance from bregma in millimeters. Animals received 10 µg of norBNI (black circles), 5 µg of norBNI (gray triangles), or saline (white circles). **B**. Mean (±SEM) CS-elicited freezing during extinction training. Each extinction block consists of 3 CS presentations. **C**. Mean (+SEM) difference scores (pre-CS freezing subtracted from CS-elicited freezing) during test in the training (A) context and the extinction (B) context. * denotes norBNI-treated animals showed significantly less freezing to the CS than saline-treated animals (p<0.01).

Experiment 1 investigated the effect of infusion of norBNI into the VH on the renewal of fear. Microinfusion cannula placements are depicted in Figure 2A. There were no significant differences in pre-CS freezing levels between norBNI- and saline-treated rats during extinction (*F_2,20_* = 0.25; *p* = 0.781) or either test session (context A: *F_2,20_* = 0.20; *p* = 0.820; context B: *F_2,20_* = 0.47; *p* = 0.632) (see Table 1). Mean (±SEM) levels of freezing to the CS across extinction are shown in Figure 2B. Analyses revealed a significant linear trend of extinction block (*F*_1, 20_ = 86.02, *p*<0.0001) confirming freezing levels decreased across extinction. There was no effect of drug (*F*_1, 20_ = 0.07; *p* = 0.794) and no effect of norBNI dose (*F*_1, 20_ = 0.04; *p* = 0.844). There was no significant two-way interaction between extinction block and drug (*F*_1, 20_ = 1.97; *p* = 0.176) or between extinction block and norBNI dose (*F*_1, 20_ = 0.61; *p* = 0.444). This indicates that there was no difference between groups in rate of extinction or overall levels of fear. This is as would be expected given that drug infusion took place subsequent to extinction training.

**Table 1:** Mean (SEM) percent pre-CS freezing for Experiment 1.

	Group		
Experimental Stage	10 µg norBNI	5 µg norBNI	Saline
Extinction	4 (2)	3 (2)	2 (1)
Test Context B	0 (0)	1 (1)	0 (0)
Test Context A	20 (3)	16 (6)	22 (5)

Performance during test was calculated as a difference score, where the percentage of freezing during the 2 min pre-CS period was subtracted from the average freezing during CS presentations. Mean (±SEM) freezing difference scores during the counterbalanced test sessions are displayed in Figure 2C. On test there was overall significantly more freezing to the CS in the training context A than the extinction context B (*F*_1, 20_ = 142.65, *p*<0.0001) confirming the renewal of fear. There was a main effect of drug on test such that overall norBNI-treated animals showed significantly less freezing to the CS than saline-treated animals (*F*_1, 20_ = 7.35, *p* = 0.013), however there was no difference in freezing levels between norBNI doses (*F*_1, 20_ = 2.11, *p* = 0.162). Furthermore, there was a significant two-way interaction between drug and context (*F*_1, 20_ = 8.70, *p* = 0.008; standardized 95% CI [−3.704, −0.014]) indicating that the difference in responding to the CS between norBNI and saline groups was significantly greater in context A than in context B. Further analyses confirmed that infusion of norBNI into the VH significantly attenuated the expression of renewal. There was no significant effect of drug (*F*_1, 20_ = 0.14, *p* = 0.712) or dose (*F*_1, 20_ = 0.19, *p* = 0.668) on CS-freezing in the extinction context B. In the training context A, animals receiving norBNI showed significantly less CS-freezing than saline controls (*F*_1, 20_ = 10.16, *p* = 0.005; standardized 95% CI [0.143, 3.366]), however there was no difference between norBNI doses (*F*_1, 20_ = 1.90, *p* = 0.183), suggesting that both doses of norBNI were effective in reducing fear renewal.

### Experiment 2: Effect of KOR antagonism in the DH on the renewal of fear

**Figure 3:**
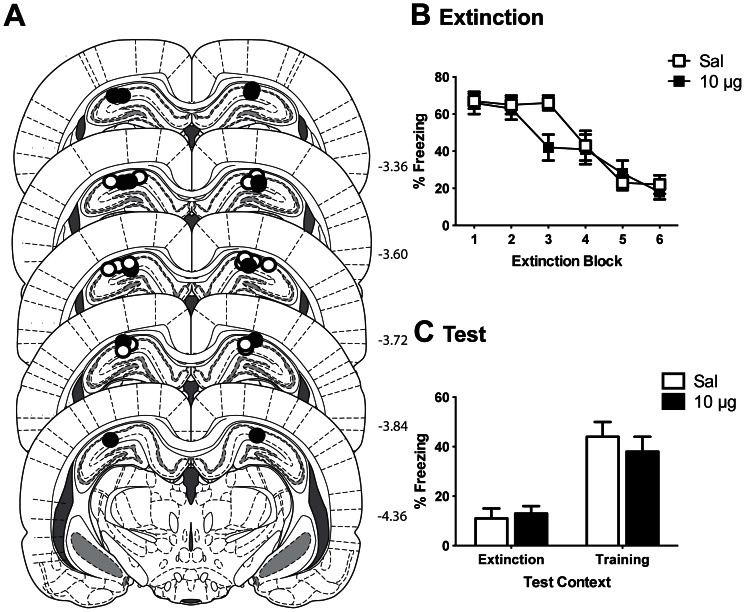
Infusion of norBNI into the dorsal hippocampus (DH) had no effect on the renewal of fear. **A**. Microinfusion cannula placements in the DH for all rats included in Experiment 2 mapped onto coronal sections from the atlas of Paxinos and Watson (2007). Numbers indicate distance from bregma in millimeters. Animals received 10 µg of norBNI (black circles) or saline (white circles). **B**. Mean (±SEM) CS-elicited freezing during extinction training. Each extinction block consists of 3 CS presentations. **C**. Mean (+SEM) difference scores during test in the training (A) context and the extinction (B) context.

The results from Experiment 1 showed that antagonizing KORs in the VH impaired the renewal of fear. Although recent research has clearly demonstrated a role for the VH in the return of fear following extinction [10], [11], the DH has also been shown to mediate renewal [5], [23]. Furthermore, the DH in the rat projects to the dorsal region of the mPFC [8], and contains KORs [24]. Therefore to investigate the neuroanatomical specificity of this role for hippocampal KORs in renewal, Experiment 2 examined the effect of infusions of norBNI into the DH on renewal. Because in the previous experiment both doses of norBNI were equally effective in attenuating renewal, here only the higher dose of 10 µg was utilized so as to reduce the number of animals used. The placement of microinfusion cannulas in the DH are depicted in Figure 3A.

There were no significant differences in pre-CS freezing levels between norBNI- and saline-treated rats during extinction (*t_13_* = 0.18; *p* = 0.68) or either test session (context A: *t_13_* = 3.29; *p* = 0.09; context B: *t_13_* = 1.09; *p* = 0.32) (Table 2). Mean (±SEM) levels of freezing to the CS across extinction are shown in Figure 3B. Analyses revealed a significant linear trend of extinction block (*F*_1, 13_ = 208.95, *p*<0.0001) confirming freezing levels decreased across extinction. Upon inspection of Figure 3B it appears as though there might be a difference in extinction between groups. However, there was no overall difference in freezing during extinction between groups (*F*_1, 13_ = 0.88; *p* = 0.365), and importantly no significant group by extinction block interaction (*F*_1, 13_ = 0.29; *p* = 0.599) indicating that there was no difference between groups in the rate they extinguished or in their overall levels of fear. Mean (±SEM) difference scores of freezing during test sessions are displayed in Figure 3C. On test there was overall significantly more freezing to the CS in the training context A than the extinction context B (*F*_1, 13_ = 45.29, *p*<0.0001) confirming the presence of renewal. There was no significant effect of group (*F*_1, 13_ = 0.20; *p* = 0.662) or group by context interaction (*F*_1, 13_ = 1.09; *p* = 0.315), indicating that infusion of norBNI into the DH had no effect on the expression of renewed fear.

**Table 2:** Mean (SEM) percent pre-CS freezing for Experiment 2.

	Group	
Experimental Stage	10 µg norBNI	Saline
Extinction	4 (2)	5 (2)
Test Context B	2 (1)	1 (0)
Test Context A	11 (4)	19 (3)

## Discussion

These experiments investigated the role of hippocampal KORs in the renewal of extinguished fear. We demonstrated that: 1) intra-VH microinfusions of the KOR antagonist norBNI significantly attenuated renewal using a within-subjects design, 2) both a 5 µg and 10 µg dose were equally effective at reducing CS-freezing in the training (A) context, and 3) intra-DH microinfusions of norBNI had no effect on the expression of renewal. Together these experiments reveal a dissociation in hippocampal contributions to renewal, where KORs in the VH, but not the DH, contribute to the renewal of extinguished fear.

These findings are consistent with previous studies demonstrating involvement of the hippocampus in fear renewal. However unlike previous studies, here we show a clear dissociation in the contribution of distinct hippocampal regions. Prior functional studies investigating the neural circuitry underlying renewal have used lesions or temporary inactivation methods to target either the DH or VH. Such studies found that inactivating either hippocampal region prevented renewal [5], [11], [23]. Clearly both the DH and VH are essential components of the circuitry mediating the return of fear following extinction, yet here we demonstrate that the contributions of these regions are distinct. Specifically, we demonstrated that only the VH involvement in renewal relies on activation of KORs, at least in part.

Recently, Orsini end colleagues [10] demonstrated that the VH mediates renewal via direct projections to the mPFC and BA. This raises the question of how KORs within the VH are acting on this circuit to mediate renewal. One possibility is through the extensive projections from the VH to the BA [6], [7]. Renewal increases Fos expression in BA-projecting neurons in the VH [10], and results in increased firing in a population of BA neurons receiving input from the VH (so called “fear” neurons; [9]). This suggests that recruitment of this VH-BA pathway is activated during renewal. Although activation of KORs in the hippocampus has been shown to inhibit excitatory transmission [25], in regions of the caudal hippocampus KORs are also located on GABAergic interneurons, with activation of KORs likely suppressing GABA release and thereby disinhibiting pyramidal neurons [26], [27]. As such, the attenuation of renewal seen here in Experiment 1 is potentially due to norBNI acting on these GABAergic interneurons to prevent the disinhibition of pyramidal neurons, reducing activation of the VH-BA pathway and thus diminishing the response of fear neurons in the BA.

Recently however, it was demonstrated that individual VH neurons send convergent projections to both the BA and mPFC, including the prelimbic cortex (PL) [28]. This is of note considering the role of the PL in renewal and the expression of conditioned fear. For example, the PL shows significant neuronal activation during renewal [29], and CS-evoked firing which correlates with learned freezing behavior [30]. Such findings raise the possibility that the attenuation of renewal by infusions of norBNI into the VH was due to simultaneous reduction in activity in both PL and BA. Of course it is important to note that KORs are widely distributed in the hippocampus, including on granule cell mossy fibres and perforant path terminals, and hence could exert numerous effects on hippocampal neurons [31]. Further studies are clearly needed to clarify the exact mechanisms through which VH KORs regulate the renewal of fear.

In light of the prior studies demonstrating involvement of the DH in the renewal of fear [5], [23], it might seem surprising that here infusion of norBNI into the DH had no significant effect. However, for a number of reasons, it seems likely that the DH and VH would perform distinct roles in fear renewal and exert these effects through distinct mechanisms. First, anatomical studies have demonstrated connectional differences between these two hippocampal regions. Specifically these studies show that the VH, but not the DH, projects directly to both the amygdala and ventral regions of the mPFC [6], [7], [8], [28], structures believed to critically mediate fear extinction and renewal [32], [33]. Second, while KOR action is predominantly inhibitory, in restricted regions of the VH KORs are located on interneurons, suggesting that the consequences of KOR activation on cell excitability also differ between the VH and DH [26], [27]. Finally, research has demonstrated dissociation in the function of these hippocampal regions. For example, the DH is critical for spatial learning and memory [34]. In contrast, the VH has been shown to regulate stress, anxiety, and other motivated behaviors [35], [36], [37], [38], which is of particular interest given the increasing evidence implicating KORs in mediating such stress, anxiety, and depressive-like behaviors [34]. Although there were slight differences in the freezing levels of saline animals across the two experiments, such small variations in responding are to be expected given that the experiments were conducted separately. Importantly however, the critical difference is not in the absolute levels of responding between experiments, but in the within-experiment comparisons, which clearly demonstrate a significant reduction in renewal by norBNI in the VH, but not the DH. It is also worth noting that although norBNI infused into the VH significantly reduced fear on test, it did not completely eliminate renewal. This suggests that the role of VH KORs is modulatory in nature, and that KOR action forms only one component of the VH contribution to renewal. However the nature of this additional mechanism, as well as the precise role of the DH in fear renewal, is yet to be determined.

Finally, our findings add to a growing body of literature suggesting KOR antagonists may have potential as a therapeutic tool for the treatment of affective disorders. Intra-hippocampal norBNI has demonstrated anti-depressant-like effects [12] and here it was shown that intra-VH norBNI reduced the renewal of fear following extinction. More generally, KOR antagonists including norBNI have been shown to impair anxiety and depressive-like behaviors in a number of paradigms [39]. These combined anxiolytic and anti-depressant properties are of particular note given that some commonly used anti-depressants can increase anxiety [40]. Although norBNI possesses anxiolytic qualities, the reduction in freezing evident here in Experiment 1 is not likely due to a general reduction in fear or possible motoric effect of the drug for a number of reasons. First, in Experiment 1 norBNI infused into the VH had no effect on freezing during the pre-CS period (Table 1), or on CS-evoked responding in the extinction context (Figure 2C). Second, it has been demonstrated that post-conditioning administration of norBNI, through either peripheral or central routes, has no effect on the expression of subsequent freezing behavior [15], [41]. Third, prior studies have shown that norBNI has no effect on general locomotor activity [13], [42]. Instead, the present results suggest that norBNI infusion into the VH acts to reduce fear to the CS which has been recovered due to a change in the environmental context, and that KOR antagonists may be helpful in promoting the long-term success of exposure-based therapies.
